# Closed-type pre-treatment device for point-of-care testing of sputum

**DOI:** 10.1038/s41598-018-34781-1

**Published:** 2018-11-07

**Authors:** Hyun-Ju Park, Ayoung Woo, Jae Min Cha, Kyu-Sung Lee, Min-Young Lee

**Affiliations:** 1 Department of Medical Device Management and Research, Samsung Advanced Institute for Health Sciences & Technology, Sungkyunkwan University, Seoul, 06351 Republic of Korea; 2 3D Stem Cell Bioprocessing Laboratory, Department of Mechatronics, Incheon National University, 119 Academy-ro, Yeonsu-gu, Incheon, 22012 Republic of Korea; 3 Department of Urology, Samsung Medical Center, Sungkyunkwan University School of Medicine, Seoul, 06351 Republic of Korea; 4 Smart Healthcare Medical Device Research Center, Samsung Medical Center, 81, Irwon-ro, Gangnam-gu, Seoul, 06351 Republic of Korea

## Abstract

The procedures and protocols for the pre-treatment of sputum specimens, mainly used for the diagnosis of pneumonia, are complex, labor intensive, and require skilled specialists working in a biosafety containment laboratory because of sample infectivity. In this study, we developed the first portable, low-power pre-treatment device that carries out all sputum pre-treatment procedures (liquefaction, homogenization, dissolution, and inactivation) in an enclosed space. Designed to simultaneously employ chemical and mechanical dissolution in the enclosed chamber, this device eliminates the risk of transmission and improves the effectiveness of sputum dissolution and pathogen detection. This device is expected to allow for the pre-treatment of infectious sputum specimens outside of a biosafety containment laboratory. Used in conjunction with automated genome extraction and detection systems, this device should make the on-site diagnosis using infectious sputum specimens possible.

## Introduction

Acute respiratory infections account for about half of all infectious diseases, with high morbidity and mortality worldwide^1^. These diseases are transmitted through the respiratory tract. In particular, Severe acute respiratory syndrome coronavirus, Middle East respiratory syndrome coronavirus, and Influenza virus are highly contagious and lethal^2,3^. Rapid diagnosis is very important to reduce the death rate and interrupt transmission. Accurate identification of the pathogens causing acute respiratory infections on site allows for rapid and appropriate antibiotic treatment instead of using a wide range of antibiotics or improper antibiotics. Such specific treatment decreases the chance of antibiotic-resistant strains of bacteria arising. Automated nucleic acid extraction and miniaturized polymerase chain reaction (PCR) systems for point-of-care testing have already been commercialized^4–7^, and isothermal nucleic acid amplification methods such as loop-mediated isothermal amplification, rolling circle amplification, and nicking enzyme amplification reaction have been developed^8–10^. Thus, it is expected that a variety of on-site genetic diagnoses will be possible. However, because of the risk of infection during the handling of specimens, infectious agent identification should be handled only by highly skilled experts in enclosed special facilities. Such facilities are only available in large hospitals or laboratories, thereby limiting the speed of acute respiratory infection diagnosis.

The sputum specimen pre-treatment procedures and protocols used to diagnose pathogens such as tuberculosis and pneumonia are complex and very labor intensive. The protocol involves liquefaction, homogenization, dissolution, and inactivation^11,12^. The pre-treatment methods for infectious sputum specimens currently used in most laboratories require not only daily preparation of reagents but also the process of opening and closing the lid for adding reagents, vortex mixing, and sedimentation using a centrifuge—all of which are done by hand^13,14^. Because there is a risk of infection during this sample pre-treatment process, the procedures must be carried out by experienced testers wearing special lab suits and masks in enclosed areas that can maintain constant negative pressure^15^. For some sputum specimens, a lysis reagent alone is not sufficient to liquefy and homogenize the sample, requiring mechanical dissolution through sonication. Gram-positive bacteria have a thicker peptidoglycan layer, and mycobacteria have complex glycolipids in their cell walls, making cell wall disruption more difficult than for other bacteria. To extract nucleic acids from these bacteria, methods such as boiling at 60–100 °C or high-energy bead beating are currently used to remove complex cell wall structures^13,16^. Therefore, the sputum pre-treatment process presently used in the laboratory is a significant obstacle to on-site diagnosis of respiratory infections. Simple paper-based sample pre-treatment systems for on-site diagnosis have been reported^17–19^, but they did not eliminate completely the risk of infection and only chemical lysis on paper may not enough to liquefy and lyse a highly viscous sputum sample. There are very few studies that developed a sputum pre-treatment system to allow for point-of-care testing of respiratory infections.

This study aims to develop a portable, low-power pre-treatment device that can carry out all of the steps of sputum treatment before nucleic acid extraction: liquefaction, homogenization, dissolution, and inactivation. In addition, we aimed to design a system that seals the sample itself during the pre-treatment process instead of sealing the person from the sample to eliminate the need for sending the sample to a central biosafety containment laboratory for analysis. To overcome problems posed by infectivity and potential cross-contamination during the reagent mixing process, the sputum pre-treatment system developed in this study was designed to allow the reagents to be mixed in a disposable, closed system. Both chemical and mechanical methods of sample dissolution were designed to take place within the closed unit, thereby ensuring promptness, convenience, and safety. To evaluate the performance of the pre-treatment device developed in this study, the effectiveness of sputum sample liquefaction and dissolution were assessed by viscosity and absorbance measurements. We also evaluated the yield of genomic DNA extraction in sputum spiked with cells and performed PCR detection using mycobacteria.

## Results and Discussion

### Fabrication and operation of the pre-treatment device

**Figure 1:**
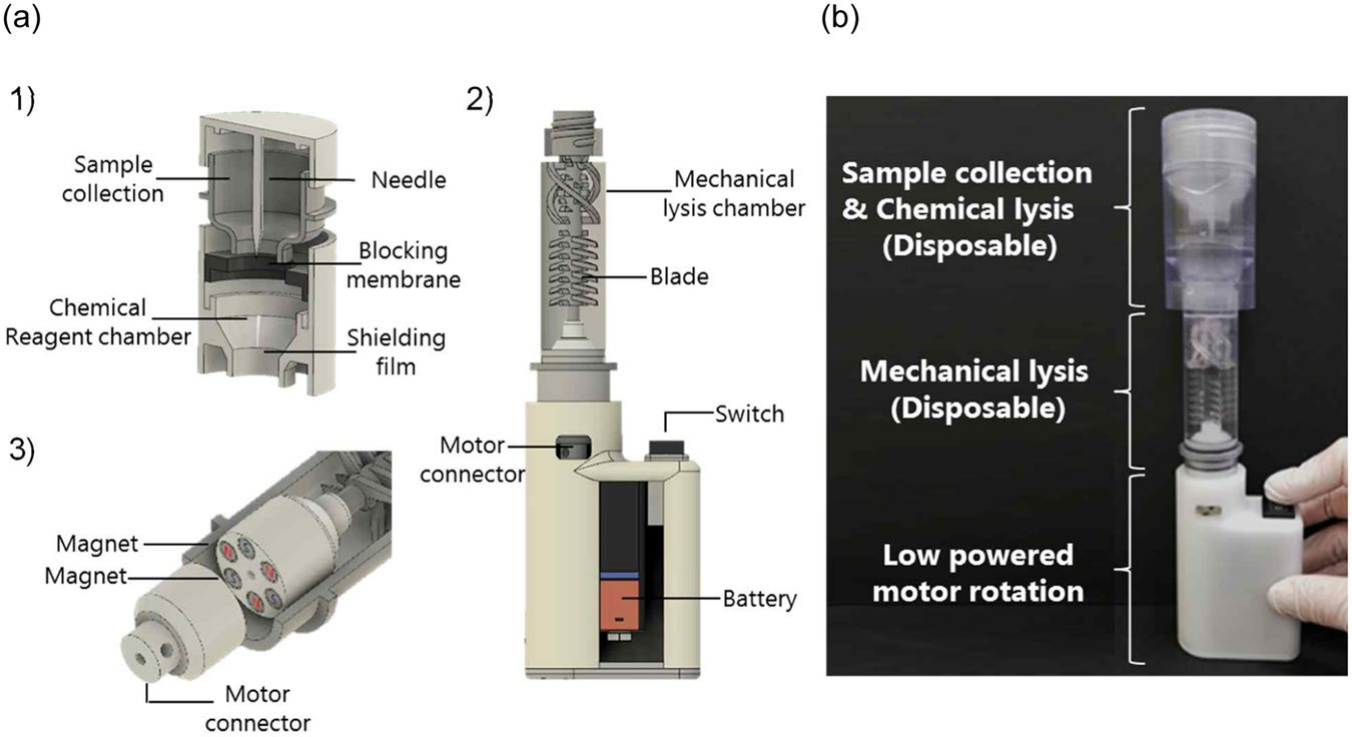
Overview of the closed-type pre-treatment device. (**a**) The device comprises 1) a sample collection and chemical reagent chamber, 2) a mechanical lysis chamber, and 3) a magnet-driven blade rotation device in an enclosed chamber. (**b**) Photograph of the actual device.

As shown in Fig. 1 and S1, the pre-treatment device developed in this study consists of a collection chamber in which the sputum sample is placed, a mechanical lysis chamber that can be connected to the collection chamber, a magnet-based rotary drive for rotating the blade in the mechanical lysis chamber, and a low-power motor for controlling the operation of the device. The sample collection and mechanical lysis chambers are designed for single use. A chemical reagent chamber separated by a blocking membrane is contained in the collection chamber. Isolation of chemical reagents is essential in devices for use in clinical practice to prevent inhalation or ingestion of the reagent. Mechanical dissolution was added to increase the dissolution efficiency and to prevent diagnostic errors related to the degree of liquefaction and homogenization of the sputum. The mechanical lysis chamber has a cylindrical shape with a rotating blade inside. Instead of connecting the blades directly to the motor, the magnets are attached to the blade and motor parts such that the magnetic rotational forces are applied to allow completely closed mechanical dissolution. The mechanical lysis chamber is detachable and disposable. The motor and the battery are portable, with low power consumption.

Operation of the device includes the following steps (Fig. S2): (1) placing the sample into the collection chamber that is connected to the mechanical lysis chamber, (2) closing the lid and pressing down on the collection chamber, and (3) connecting to the motor rotation part and pressing the switch. When the lid is closed, the sample is mixed with the chemical reagent by breaking the membrane with the needle. After pressing down on the collection chamber, the shielding film under the reagent chamber is broken, allowing the mixture to enter the mechanical lysis chamber. When the switch is pressed, the blade rotates in a blender. (4) Liquefied, homogenized, and inactivated sputum specimens in the closed device are then (5) extracted with a syringe through a rubber membrane in the lid of the collection chamber for antigen or nucleic acid assays.

### Sputum liquefaction

**Figure 2:**
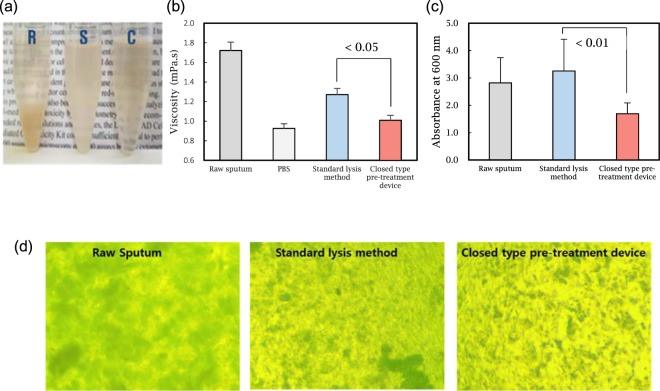
Sputum liquefaction. (**a**) Photograph comparing sputum samples treated as follows: R, unliquefied raw sample; S, liquefied using the standard lysis method; C, liquefied using the closed type pre-treatment device. (**b**) Sample viscosities. (**c**) Sample absorbance at 600 nm. (**d**) Microscopic images of samples.

The high viscosity and lumpy nature of sputum are major hindrances to both antigen- and nucleic acid-based diagnostics. Liquefaction and homogenization of sputum are essential for proper diagnoses. The degree of liquefaction of sputum specimens pretreated with our device was compared with that of the standard method by vortex mixer used in clinical evaluations. The photographs in Fig. 2a show the difference in appearance between non-liquefied raw sputum samples, liquefied sputum samples by the standard lysis method using a vortex mixer, and our pre-treatment device. The non-liquefied sputum had a mass of precipitated viscous materials. The sputum sample liquefied using a vortex mixer appeared to be loosened, but small lumps were still present, and the solution was opaque. In contrast, the sputum sample liquefied using the pre-treatment device had few lumps and was transparent enough to allow viewing of print through the sample. To quantitatively evaluate the ability of liquefaction through mechanical grinding of the pre-treatment device developed in the present study, the viscosity, absorbance of the solution (turbidity), and particle size in the sputum were investigated. Fig. 2b shows the viscosity of sputum before and after liquefaction. The non-liquefied sputum sample (diluted 1:1) had a high viscosity of about 1.7 mPa·s, and the sputum sample liquefied using the standard method had a relatively low viscosity of about 1.27 mPa·s. However, the viscosity of the sputum sample liquefied using our device was about 1.01 mPa∙s, which was close to the viscosity of PBS (0.93 mPa∙s) as measured in this study. The liquefied sputum sample had a relatively uniform viscosity, indicating that the mechanical liquefaction process in the pre-treatment device effectively breaks down the viscous material, with greater efficiency. Absorbance can be used to compare the turbidity of the solution. As the sputum breaks and liquefies, the solution becomes transparent and absorbance at 600 nm decreases. As shown in Fig. 2c, the absorbance at 600 nm of sputum liquefied using the pre-treatment device was 1.92 times lower than that using the standard method. Particle size can also be used to assess the degree of brokenness of the sputum. Microscopic observation of the sputum samples revealed large particles in raw sputum and smaller particles in that liquefied using the standard method (Fig. 2d). In contrast, much smaller particles were seen in the sputum liquefied using the pre-treatment device. Taken together, we confirmed that the liquefaction efficiency of the sputum using the pre-treatment device is superior to that using the standard liquefaction method using vortexing.

### Cell viability

**Figure 3:**
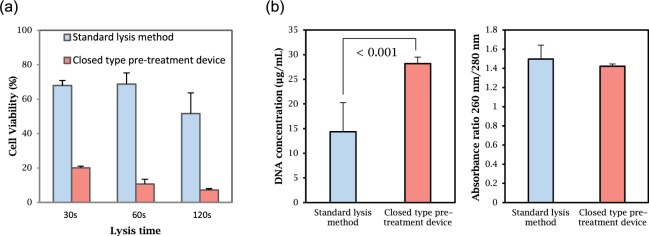
Cell viability and DNA extraction. (**a**) Cell viability. (**b**) Yield of DNA extracted (left) and DNA purity (right) using the standard lysis method and closed-type pre-treatment device.

Infectious specimens require a process to kill and inactivate bacteria or viruses to eliminate the risk of transmission prior to diagnosis. The Cepheid GeneXpert MTB/RIF sample inactivation process recommends a 15 min incubation after adding the chemical reagent for inactivation, but a recent report suggests that 60 min of incubation time is required for complete inactivation of *mycobacterium tuberculosis* (MTB)^13^. We compared cell viability after liquefaction by the standard method and our pre-treatment device using dithiothreitol (DTT) solution. After pre-treatment for 30, 60, or 120 s, tests using MTS solution showed that cell viability decreased with increasing treatment time and was significantly lower after inactivation in the pre-treatment device than with vortex mixing for all three treatment times (Fig. 3a). Nearly all of the cells died when treated for 120 s. These results indicate that mechanical dissolution in the pre-treatment device provides more effective DTT treatment and cell wall disruption than does the standard vortex mixer method. This is thought to be a synergistic effect due to mechanical lysis. The use of the lysis solution may lead to cell death much more quickly, and rapid on-site inactivation of the sample will be possible.

### Cellular DNA extraction

The efficiency of DNA extraction from sputum specimens spiked with cells was investigated (Fig. 3b). The standard lysis method yielded about 14.4 µg/mL, whereas the pre-treatment device yielded about 28.2 µg/mL, nearly double the amount. This higher yield likely resulted from the greater extent of sputum liquefaction and cell lysis provided by the pre-treatment device. The 260/280 ratio, representing the purity of the extracted DNA, was 1.49 by the standard lysis method and 1.42 by the pre-treatment device, with no significant difference. This is because the sputum contains a large amount of protein components; therefore, the extracted sample is considered to have somewhat low 260/280 ratios. However, even though the 260/280 ratio by the standard lysis method and the pre-treatment device was similar, it was confirmed that the concentration of DNA extracted from the liquefied sputum by the pre-treatment device was nearly twice as high as that by the standard lysis method.

### qPCR for mycobacterial detection

**Figure 4:**
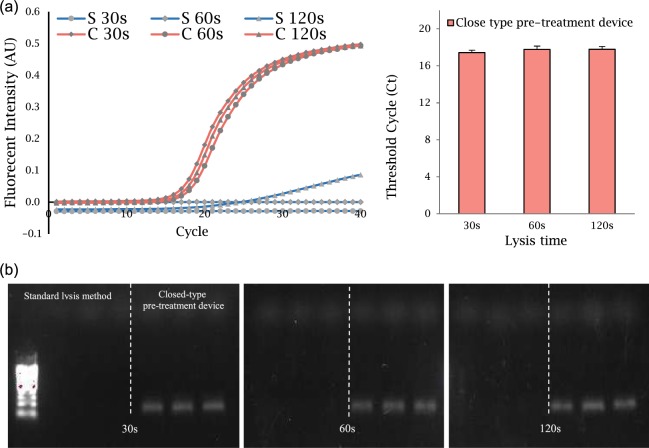
Comparison of mycobacterial DNA extraction and amplification between treatment methods. (**a**) Amplifiable DNA quantified by qPCR (left) and Ct value (right) using standard lysis method (S) and closed-type pre-treatment device (C) (operating time: 30, 60, or 120 s). (**b**) Agarose gel electrophoresis of amplified DNA. The full-length gel is presented in Supplementary Figure S3.

Mycobacteria are difficult to lyse, requiring different reagents than those used for extracting DNA from viruses and Gram-negative bacteria; in addition, further lysis methods such as boiling or high-energy bead beating are required^13^. We compared the dissolution rates of the nontuberculous *M*. *fortuitum* between vortexing and treatment in our device. As shown by the amplification plot in Fig. 4a, DNA amplification was observed only in the group treated using the pre-treatment device. This result suggests that the mechanical lysis process in the pre-treatment device disrupts the mycobacterial cell wall, allowing for effective DNA extraction. The threshold cycle (Ct) of PCR amplified by lysis time of the closed-type pre-treatment device was 17.726 for 30 s, 18.13f2 for 60 s, and 18.141 for 120 s (Fig. 4b). There is little difference in the Ct values, and it can be concluded that a lysis time from 30 s to 120 s does not have a large effect on PCR amplification. From this result, it can be stated that a lysis time of 30 s is enough for the DNA release from *M. fortuitum*. The slightly increase in the Ct value over the lysis time may be due to damage to the DNA by the lysis reagent^20^. Electrophoresis results confirm that no DNA was amplified in samples treated by the vortex method, whereas all the bands were present after treatment in the pre-treatment device. The amplified gene of 173 bp length was confirmed by the electrophoresis and corresponding to 100–200 bp (Fig. 4c).

## Conclusion

For the diagnosis of acute respiratory infectious agents from sputum, we developed a portable, closed-type pre-treatment device to allow for safe sample treatment without the use of biosafety hoods. In this device, mixing of chemical reagent and mechanical dissolution can be performed without opening the lid after sputum collection. The mechanical dissolution is powered by a low-power battery, which is inexpensive, simple, and portable. Even in resource-limited settings, samples can be processed quickly, without risk of infection, using our system.

We found that the closed-type pre-treatment device is much more efficient than the conventional vortex mixing with respect to sputum liquefaction, homogenization, lysis, and inactivation. This device also effectively detects DNA from mycobacteria which are difficult to lyse. This treatment takes place in a small, enclosed device and requires no heating, making it suitable for on-site analysis. We are considering a new design for the sputum collecting unit to address patient convenience and plan to conduct follow-up studies to optimize the conditions for screening for tuberculosis, MERS, etc. When used in conjunction with automated extraction and amplification systems, this device is expected to make the on-site diagnosis of acute respiratory infections possible.

## Methods

### Device fabrication and operation

The closed pre-treatment device was designed using computer-aided design software. The specimen collector, chemical reagent chamber, and mechanical dissolving portions of this device were made by 3D printing using polycarbonate as a transparent material, and the rotating parts of the motor, including the magnet, were manufactured by the cutting method using acetal. A small, round magnet was mounted for the blade rotation. A small 9 V motor and 9 V battery were used. The specimen is placed in the specimen collector of the device, the lid is closed, and the motor is plugged in and turned on by pressing the switch.

### Sputum sample collection

The sputum used in the experiment was collected from healthy people over 18 years old who were not infected with pathogens and who showed no abnormal symptoms. We fully explained this study to the participants, obtained their informed consent for study participation, and collected the sputum. The collected sputum samples were used anonymously. We performed the collection and pre-treatment experiments of sputum according to the guidelines of Samsung Medical Center after receiving approval for the experimental protocol from the Institutional Review Board of Samsung Medical Center. The sputum was collected in a 50 mL conical tube (Falcon, Thermo Fisher Scientific, NC, USA) and stored at −80 °C until use. Sputum samples of similar volume and state were chosen for the following experiments.

### Sputum liquefaction

The standard liquefaction method for sputum is as follows. The sputum sample is uniformly mixed with an equal volume of 0.1% dithiotreitol (DTT) using a vortex mixer for 30 s and is incubated at room temperature for 15 min^11^. In the present study, the sputum sample and 0.1% DTT solution were mixed and vortexed for 30 s without incubation time for 15 min. The sputum liquefaction efficiency of the closed-type pre-treatment device was investigated by comparison with that of the standard method. Distilled water (1 mL) containing 0.1% dithiothreitol (DTT, Sigma Aldrich, St. Louis, MO, USA) was added to the chemical reagent chamber, which is separated from the sputum collector by a rubber membrane. The sputum sample was placed in the sputum collector, the lid was closed, and the switch was pressed to rotate the blade for 30 s. DTT allows the sputum protein to be liquefied^21,22^. To compare the degree of liquefaction of the sputum, samples were placed into 15 mL tubes and photographed with an ordinary camera. From each sample was taken 100 μL to place in a well of a 96-well plate. These samples were observed under a microscope. The viscosity of the liquefied sputum was measured using the Mems & New paradigm for flow management (Rheosense, Inc., San Ramon, CA, USA) (Average *n* = 3–4 independent experiments per group). After each sample measurement, the device was cleaned more than 2 times in washing mode to prevent sample contamination. The data were analyzed using the Micro-ViSC program. The absorbance of each sample was measured at 600 nm using an Eppendorf BioSpectrometer kinetic (Eppendorf, Hamburg, Germany) (*n* = 5–6 independent experiments per group). If the value was higher than the maximum value of 3 that can be displayed on the machine because of high absorbance, it was recorded as 3.

### Cell viability

The effect of mechanical dissolution using the pre-treatment device was examined. PC 12 cells, a cell line derived from a pheochromocytoma of the rat adrenal medulla (Samsung Medical Center, Seoul, Korea), were dispersed in 1 mL of phosphate-buffered saline (PBS, pH 7.4) to 2.5 × 10^5^/mL and 1 mL of 0.1% DTT solution and treated in the same manner as for sputum liquefaction described above. The liquefaction times were set to 30, 60, and 120 s. Then, a 100-μL aliquot of each pretreated sample was dispensed into one well of a 96-well plate, to which 100 μL of RPMI Medium 1640 (GIBCO, Waltham, MA, USA) was added. CellTiter 96 AQueous One Solution Cell Proliferation Assay reagent (20 μL) (MTS, Promega Corporation, USA) was added, and the absorbance was measured at 490 nm after 3 h incubation. Cell viability was calculated using untreated cells as a control.

### Genomic DNA extraction

The sputum sample was spiked with 20 μL of 5 × 10^6^ PC 12 cells/mL in approximately 1 mL of sputum sample, and the amount of DNA extracted was compared. One milliliter of lysis buffer (buffer 1, MagListo Kit, Bioneer, Daejeon, Korea) was pre-loaded into the chemical reagent chamber. After adding the cell-spiked sputum sample, the lid was closed, and the device was allowed to run for 30 s. For comparison, 1 mL of lysis buffer was added to the sputum sample and mixed with a vortex mixer for 30 s. After dissolution, the DNA was extracted according to the protocol of the MagListo Kit (Bioneer). The final volume of elution buffer was 50 μL. The amount of extracted DNA was measured using Nano drop (ND-2000, Thermo Scientific, Waltham, MA, USA).

DNA extraction experiments were performed using *M. fortuitum* (Samsung Medical Center), a nontuberculous mycobacterium. To 500 μL of *M*. *fortuitum* suspension (OD600 = 0.185) was added 500 μL of lysis buffer (Qiagen, Hilden, Germany), followed by mixing in the pre-treatment device for 30, 60, or 120 s. Vortex mixing was also performed for 30, 60, or 120 s. Except under lysis conditions, genomic DNA was extracted using DNeasy Blood & Tissue Kit (Qiagen) according to the kit protocol and was then eluted with 200 μL of the kit’s elution buffer.

### Quantitative PCR

Quantitative PCR (qPCR) was performed to compare the amounts of DNA extracted from *M*. *fortuitum*. The forward and reverse primers used for qPCR were synthesized as 5′-TCACCTGATCTGCACATAATGT-3′ and 5′-AGCACCTCATGCGACTT-3′, respectively (Bioneer, Korea)^23^. To sterilized PCR tubes was added 10 μL of genomic DNA extracted from *M*. *fortuitum* by the standard method and the pre-treatment device as mentioned above, primers (final concentration, 0.5 μM), and SYBR Green Realtime PCR Master Mix (20 μL total reaction volume) (QPK-201, Toyobo, Japan). Real-time PCR was performed using the QuantStudio 6 Flex Real-Time PCR System (Life Technologies, Carlsbad, CA, USA) coupled with QuantStudio real-time PCR software v1.1. The PCR amplification conditions were as follows: 95 °C pre-denaturation step for 1 min, 15 s denaturation step at 95 °C, and 40-step extension at 60 °C for 40 s. The amplified PCR products were identified by electrophoresis using 2% agarose gel.

## Electronic supplementary material

Supplementary Information
