# Author Correction: Closed-type pre-treatment device for point-of-care testing of sputum

**DOI:** 10.1038/s41598-018-36359-3

**Published:** 2018-12-17

**Authors:** Hyun-Ju Park, Ayoung Woo, Jae Min Cha, Kyu-Sung Lee, Min-Young Lee

**Affiliations:** 1 Department of Medical Device Management and Research, Samsung Advanced Institute for Health Sciences & Technology, Sungkyunkwan University, Seoul, 06351 Republic of Korea; 2 3D Stem Cell Bioprocessing Laboratory, Department of Mechatronics, Incheon National University, 119 Academy-ro, Yeonsu-gu, Incheon 22012 Republic of Korea; 3 Department of Urology, Samsung Medical Center, Sungkyunkwan University School of Medicine, Seoul, 06351 Republic of Korea; 4 Smart Healthcare Medical Device Research Center, Samsung Medical Center, 81, Irwon-ro, Gangnam-gu, Seoul, 06351 Republic of Korea

Correction to: *Scientific Reports* 10.1038/s41598-018-34781-1, published online 07 November 2018

In the Supplementary Information file originally published with this Article, the author Jae Min Cha was omitted. This error has been corrected in the Supplementary Information that now accompanies the Article.

