# Supplementary material for: Closed-type pre-treatment device for point-of-care testing of sputum
Source: Sci Rep. 2018 Nov 7;8:16508. doi: 10.1038/s41598-018-34781-1 (PMC6220321; doi:10.1038/s41598-018-34781-1)
Supplement: Supplementary file 1 — Supplementary Information [file 41598_2018_34781_MOESM1_ESM.docx]

Supplementary Information

**Closed-type pre-treatment device for point-of-care testing of sputum**

**
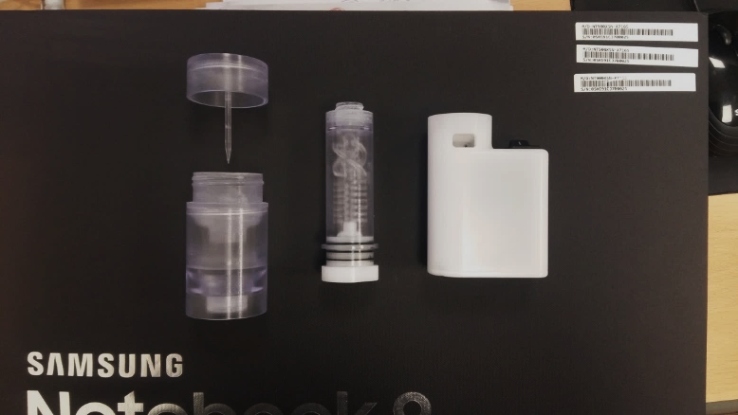
**
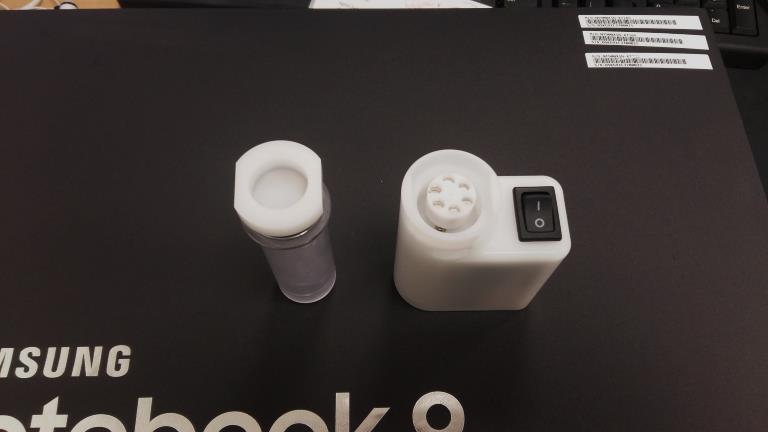
 Hyun-Ju Park,^1†^ Ayoung Woo,^1†^ Jae Min Cha,^2^ Kyu-Sung Lee,^1,3,4*^ Min-Young Lee^1,4^*

(b)

(a)

**
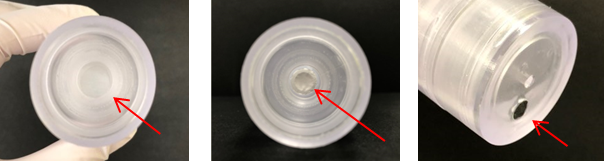
**

(c)

**Figure S1. Each parts of the device.** (a) Sample collection and chemical lysis chamber (left), mechanical lysis chamber (middle), and motor rotation part (right). (b) Top view of inverted mechanical lysis chamber (left) and motor rotation part (right). (c) Blocking membrane between sample collection and chemical lysis chamber (left), shielding film under the chemical lysis chamber (middle), and rubber membrane in the lid for sample extraction (right).


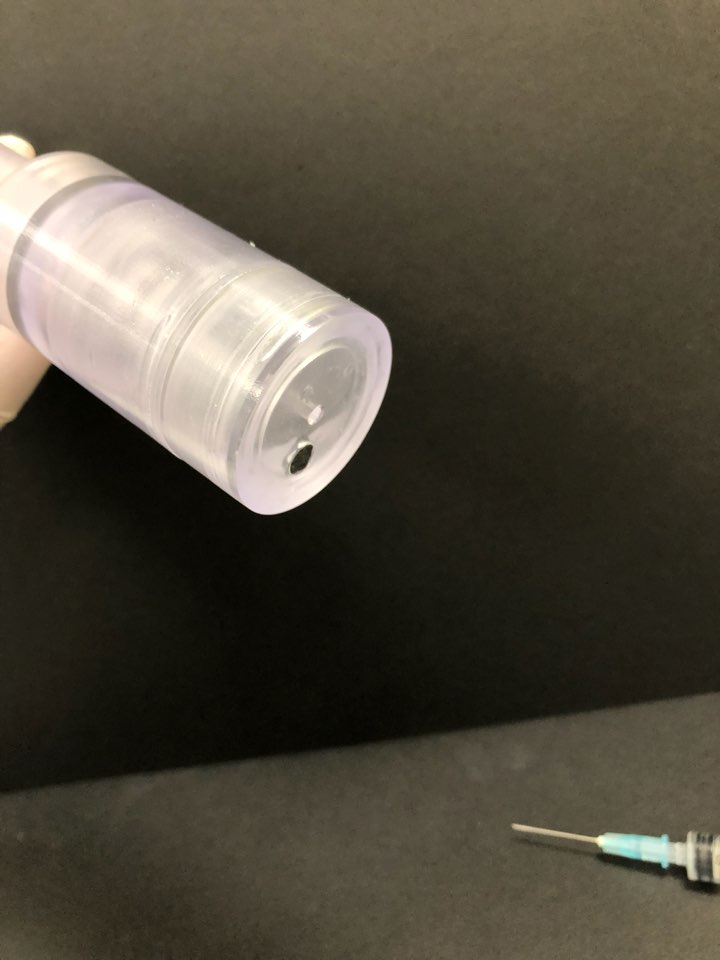

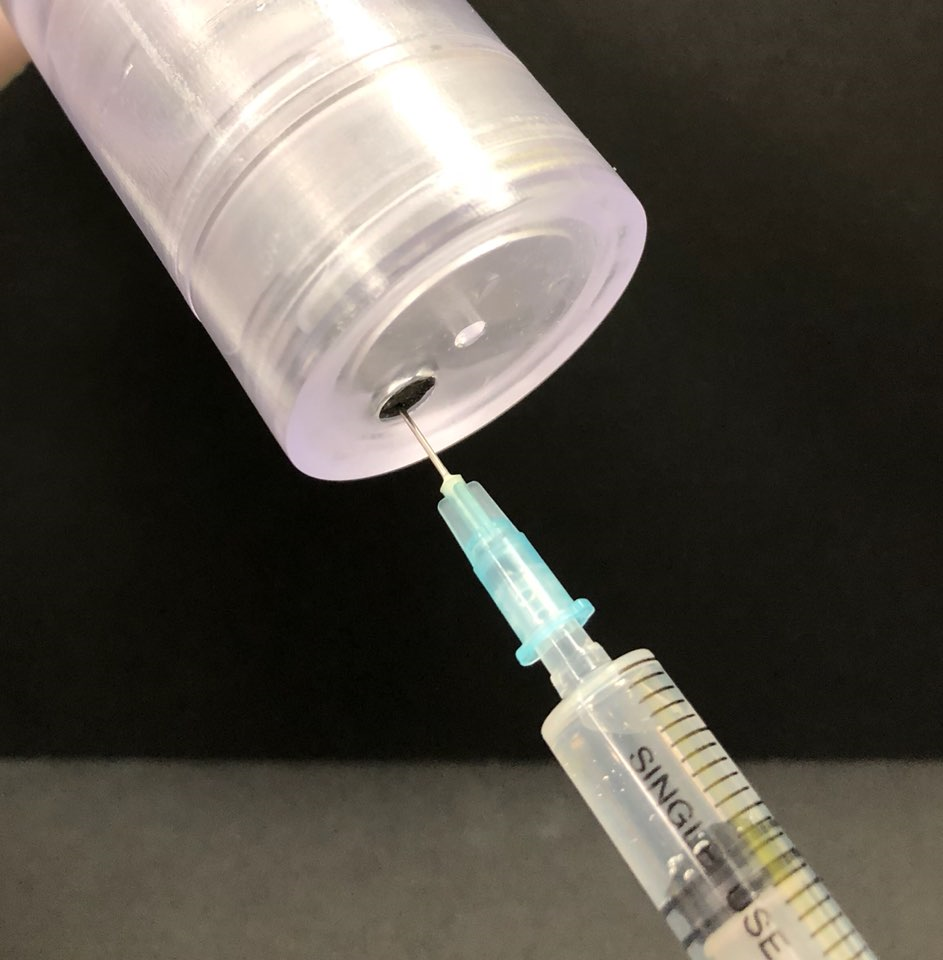

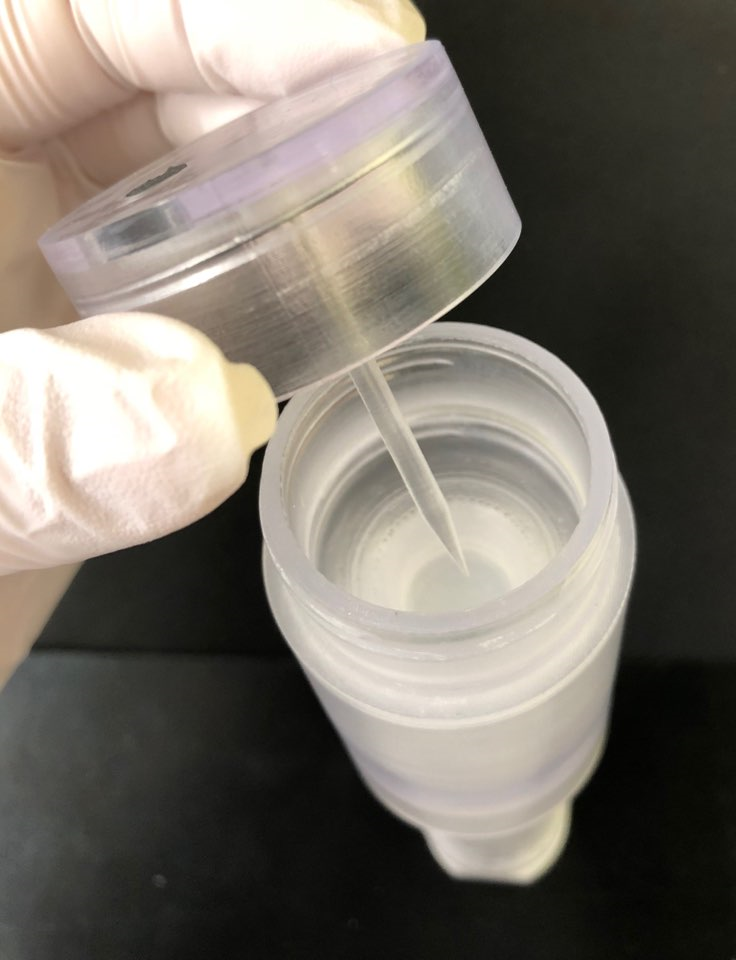

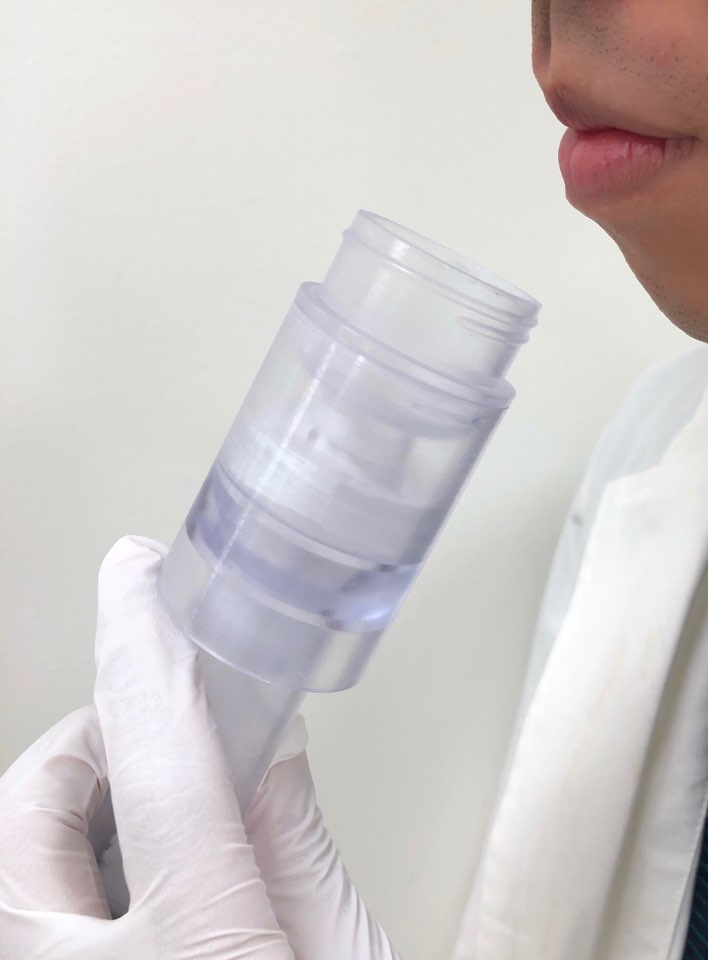

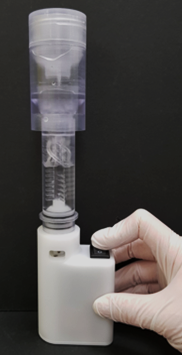


1

2

3

4

5

**Figure S2. Whole operation processes for pre-treatment of sputum sample.** 1) Sample collection, 2) Close the lid, 3) Connect to the motor and press the switch, 4) Separate from motor and turn upside down, and 5) Extract the liquefied and lysed sample using syringe.


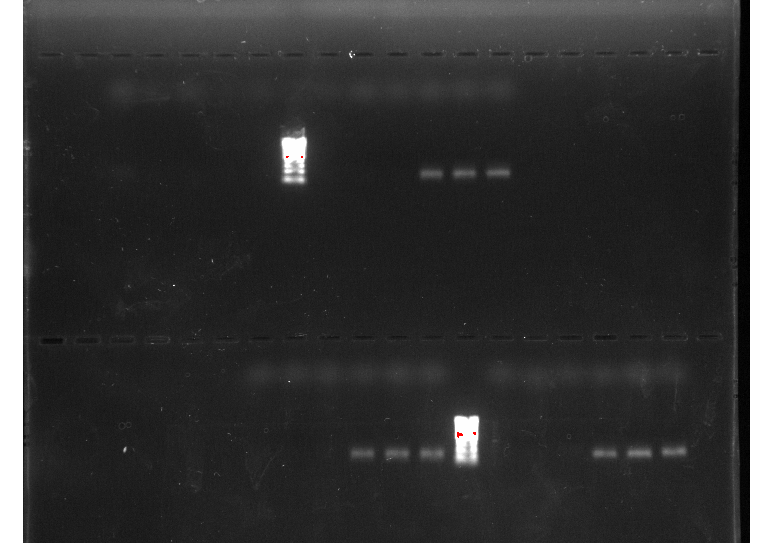


120s

60s

30s

100bp

ladder

Pre-treatment device (120s)

Closed type

method (120s)

Standard lysis

Standard lysis

100bp

ladder

method (60s)

Closed type

Pre-treatment device (60s)

Closed type

Pre-treatment device (30s)

Standard lysis

method (30s)

**Figure S3. Full-length gel electrophoresis of Figure 4b.**
